# Plant–soil feedback and plant invasion: effect of soil conditioning on native and invasive *Prosopis* species using the plant functional trait approach

**DOI:** 10.3389/fpls.2024.1321950

**Published:** 2024-01-16

**Authors:** Hamada E. Ali, Ahmed M. Al-Wahaibi, Muhammad Shafiq Shahid

**Affiliations:** ^1^Department of Biology, College of Science, Sultan Qaboos University, Muscat, Oman; ^2^Life Science Unit, College of Science, Sultan Qaboos University, Muscat, Oman; ^3^Department of Plant Sciences, College of Agricultural and Marine Sciences, Sultan Qaboos University, Muscat, Oman

**Keywords:** invasional meltdown, invasion process, Oman, *Prosopis juliflora*, *Prosopis cineraria*, plant-soil feedback

## Abstract

**Introduction:**

Invasive species have been identified as a major threat to native biodiversity and ecosystem functioning worldwide due to their superiority in spread and growth. Such superiority is explained by the invasional meltdown phenomena, which suggests that invasive species facilitate the establishment of more invasive species rather than native species by modifying the plant-soil feedback (PSF).

**Methods:**

We conducted a two-phase plant-soil feedback experiment using the native Prosopis cineraria and the invasive Prosopis juliflora in Oman. Firstly, we conditioned the soil by planting seedlings of native species, invasive species, native and invasive species “mixed”, and unconditioned soil served as a control. Secondly, we tested the feedback of these four conditioned soil on the two species separately by measuring the productivity (total biomass) and the performance in the form of plant functional traits (plant height, specific leaf area (SLA), leaf nitrogen content (Nmass), leaf carbon content (Cmass) and specific root length (SRL) of native and invasive species as well as the nutrient availability in soil (soil organic carbon (SOC) and soil total nitrogen (STN)).

**Results and discussion:**

We found that the native species produced more biomass, best performance, and higher SOC and STN when grown in soil conditioned by native species, additionally, it gave lower biomass, reduced performance, and lower SOC and STN when grown in the soil conditioned by invasive and mixed species. These results suggest negative PSF for native species and positive PSF for invasive species in the soil conditioned by invasive species, which can be considered as red flag concerning the restoration of *P. cineraria* as an important native species in Oman, as such positive PSF of the invasive species *P. juliflora* will inhibit the regeneration of *P. cineraria*.

## 1 Introduction

The problem of invasive plants has become a major issue for many natural ecosystems (Vilà et al., 2011; Pyšek et al., 2012). These invasive plants have been able to successfully spread due to their superior traits in comparison to those of native species, such as high reproductive rates and fast growth (van Kleunen et al., 2010), which can lead to a reduction in native biodiversity and consequently ecosystem functioning (Pejchar and Mooney, 2009; van Kleunen et al., 2010). Furthermore, the presence of invasive plants can facilitate the establishment of further invasive species, a phenomenon known as invasional meltdown (Simberloff and Von Holle, 1999), which suggests that invasive plants can enhance subsequent invasions in the current invaded ecosystems (Shen et al., 2023).

Recent research has indicated that belowground processes, such as plant–soil feedback (PSF), play a vital role in increasing the rate of plant invasions (Fahey and Flory, 2021; Thakur et al., 2021). PSF refers to the idea that plants can impact future plant growth by changing the biotic and abiotic structure of the soil in which they grow (van der Putten et al., 2013). Such feedback can be negative “competitive” when a species grows better in soil trained by different species (Mangan et al., 2010; Bennett and Klironomos, 2019); positive “facilitative,” which happens when growth is increased in soil trained by the same species (van der Putten et al., 2013; Thakur et al., 2021); or neutral when species exhibit no differences when grown in soil trained by the same or different species (Cushman and Gaffney, 2010). Invasive plant species can alter PSF by changing the composition and intensity of the soil microbiome, which consequently affects the performance of native plants (Reinhart and Callaway, 2006; Allen et al., 2018) and may make the ecosystem more susceptible to invasion (Chen and van Kleunen, 2022b). Recent studies suggest that the presence of invasive plants can result in positive PSF, which impairs the growth of native species and promotes the growth of more invasive species (Chen and van Kleunen, 2022b).

As invasional meltdown and PSF can be explained by plant functional traits, we selected six plant parameters that can capture differences in the production and performance of plants, namely, total biomass, plant height, specific leaf area (SLA), leaf nitrogen content (N_mass_), leaf carbon content (C_mass_), and specific root length (SRL). These indexes might give further understanding on how invaders can facilitate the success of more invaders. Biomass and plant height reflect the efficiency of plants in nutrient acquisition and competitive strength (Moles et al., 2009); SLA is related to plant growth rates (Knops and Reinhart, 2000; Garnier et al., 2001); leaf nitrogen reflects the photosynthesis rates as most N in the leaves is located in ribulose-1,5-bisphosphate carboxylase/oxygenase (RuBisCo), the main enzyme of carbon fixation (Yang et al., 2020); and leaf carbon content is connected to nutrient acquisition (Xing et al., 2021). We measured SRL, which is the ratio between the length and mass of roots and considered an important trait in expressing the plant efficiency in nutrient and water uptake (Ostonen et al., 2007). Moreover, we selected two soil parameters that can reflect differences in soil nutrient availability, soil organic carbon (SOC) and soil total nitrogen (STN), as both reflect soil fertility, soil quality, and consequently plant growth (Crowther et al., 2016).

In Oman, invasive plant species are posing a serious risk to ecosystems and biodiversity (Patzelt et al., 2022). *Prosopis juliflora* (Sw.) DC. is among the invasive plants that are currently posing a severe threat to the natural ecosystem all over the Sultanate of Oman, and it has become difficult to manage due to the lack of knowledge about how these plants are so successful invaders (Patzelt et al., 2022). *P. juliflora*, which is commonly called mesquite, is an evergreen tree natively found in central and northern South America; as it has a high ecological adaptability, it is vigorously invading plenty of natural areas in Oman due to its ability to adapt and grow under harsh arid environmental conditions (Shackleton et al., 2015). Additionally, it is suggested that *P. juliflora* affects the nearby native species due to its ability to produce allelopathic chemicals that limit the growth of other native species and alter soil nutrients (El-Keblawy and Abdelfatah, 2014; Shackleton et al., 2015; Bibi et al., 2023). *P. juliflora* competing with other native species, e.g., *Prosopis cineraria* (L.) Druce trees, which is one of the species native to Oman and is a valuable species that helps in improving soil nutrients, provides fodder for domestic animals, has a variety of medicinal and cosmetic uses, and is a potential species for biodiesel production (Pickering and Patzelt, 2008; Giustra et al., 2022; Patzelt et al., 2022). Understanding such interplay between the invasion process and PSF will help in managing this invasive species in Oman.

The current study is one of the few studies investigating the factors that control the successful spread of the invasive plant species *P. juliflora* in Oman in a way to control its negative impacts on the ecosystem level. More specifically, we asked the following questions:

1. How does an invasive species alter plant–soil feedback and soil properties (e.g., nutrient availability) in comparison to native plant species?
2. To what extent do these altered soil properties affect the performance and production of native plant species?

## 2 Materials and methods

### 2.1 Experimental setup

**Figure 1:**
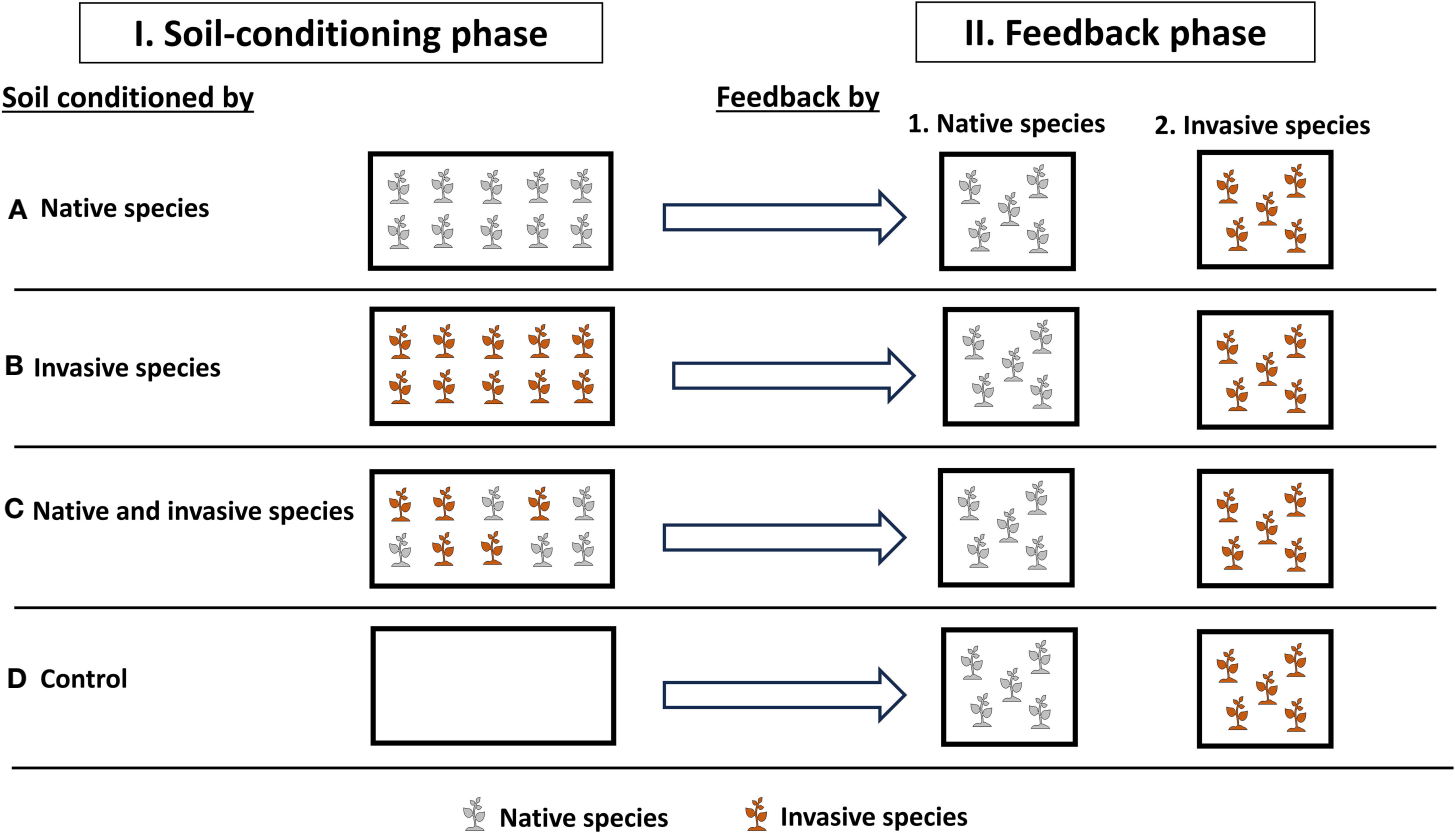
Experimental design to investigate the effects of soil conditions (native only, invasive only, mixed “native and invasive,” and control “unconditioned soil”) and feedback by (native and invasive species) on the production and performance of the native species *P. cineraria* (gray) and invasive species *P. juliflora* (orange) (*n* = 10 pots per treatment in the soil-conditioning phase and *n* = 5 pots per treatment per species in the feedback phase).

To test how soil conditioning with native and invasive species will affect the plant performance and production and soil properties, we ran a two-phase experiment that included a soil-conditioning phase and a feedback phase in the greenhouse at the Sultan Qaboos University, Muscat, Oman (23°36′0.95″N, 58°10′5.29″E) (**Figure 1**). To carry out this study, we used two species, *P. cineraria* (L.) Druce, which is a plant native to Oman, and *P. juliflora* (Sw.) DC., which is an invasive species in Oman and is currently competing with *P. cineraria* and other native species (**Supplementary Figure 1**) (Pickering and Patzelt, 2008; Patzelt et al., 2022). Seeds were collected directly from the field, and the two species have comparable germination and establishment rates.

#### 2.1.1 Soil-conditioning phase

On 4 September 2022, we sowed seeds of *P. cineraria* and *P. juliflora* for the soil-conditioning phase in separate trays (60 cm × 30 cm × 3 cm) filled with universal potting soil (Potgrond, Van Egmond Potgrond B.v., Rijnsburg, Holland) (**Figure 1**). These were placed in the greenhouse under temperature control at 25°C during daytime and 16°C during nighttime and watered twice a week.

On 2 October 2022, seedlings of both *P. cineraria* and *P. juliflora* were transplanted into 40 pots (length = 50 cm, width = 21.5 cm, and height = 16 cm) filled with pre-sieved soil collected by digging from the Sultan Qaboos University Botanic Garden. These pots were then separated into four main treatments to condition soil (10 pots each) (control, native only, invasive only, and mixed “native and invasive”) in which we transplant 10 seedlings of *P. cineraria* native species only per pot (*n* = 10 pots), 10 seedlings of *P. juliflora* invasive species only per pot (n = 10 pots), and 10 seedlings of *P. cineraria* native and *P. juliflora* invasive species (5 seedlings per pot) (*n* = 10 pots), and control (*n* = 10 pots), which received no seeds (**Figure 1**). These pots were randomly placed in the greenhouse and watered twice a week for 10 weeks. We replaced seedlings that died within the first 2 weeks of the soil-conditioning phase. Seedlings of other species grown within the study plots were removed continuously during the experiment.

On 9 December 2022, we harvested the plants and soil by cutting the above- and belowground biomass and sieving the soil through a 5-mm sieve to remove any plant materials. These soils were kept separately based on the treatment at 4°C to be used in the feedback phase.

#### 2.1.2 Soil-feedback phase

On 18 December 2022, we sowed seeds of *P. cineraria* and *P. juliflora* to be used in the feedback phase exactly under the same conditions as the soil-conditioning phase (see above). On 15 January 2023, we prepared pots (length = 11 cm, width = 11 cm, and height = 12 cm) containing soil conditioned by control soil (*n* = 10 pots), native only (*n* = 10 pots), invasive only (*n* = 10 pots), and mixed (*n* = 10 pots). Then, we transplanted five seedlings of *P. cineraria* and five seedlings of *P. juliflora* into each conditioned soil separately, resulting in a total of 40 pots (2 species × 4 soil-conditioning treatments × 5 replicates) (**Figure 1**).

All pots were randomly allocated within the greenhouse under temperature control at 25°C during daytime and 16°C during nighttime, watered twice a week, and rearranged every 2 weeks. We did not use any fertilizers during the two phases of the experiment to mimic field conditions. The feedback phase lasted for 10 weeks, and then, we harvested the above- and belowground biomass for each individual at each treatment, and roots got washed away from any remnant soil.

### 2.2 Plant and soil measurements

By the end of the feedback phase on 26 March 2023, the selected above- and belowground traits [plant height, SLA, leaf dry matter content (LDMC), N_mass_, C_mass_, and SRL] were measured following standardized protocols (Pérez-Harguindeguy et al., 2013) on each individual within each pot to account for intraspecific trait variability (Albert et al., 2012; Ali et al., 2017). Plant height (cm) was measured using a meter. SLA was measured on three healthy and fully developed leaves for each individual in each pot as a ratio between leaf area (LA, mm^2^) and dry mass (mg) expressed as mm^2^ mg^−1^. LA was measured digitally by analyzing scanned digital images using the Easy Leaf Area software (Easlon and Bloom, 2014). The leaves were weighed to record the fresh mass and subsequently oven-dried at 70°C for 48 h and weighed again to assess the leaf dry mass (mg). Finally, the LA was divided by the leaf dry weight to calculate SLA. Additionally, we measured the leaf nitrogen and carbon percentages (N_mass_ and C_mass_) on the same oven-dried leaves that were used for measuring SLA as percentage of dry mass in 0.020 g of the milled and dried leaf tissue by using a PerkinElmer 2400 CHNS organic elemental analyzer.

For biomass harvest, the plants were cut at the soil surface and the whole root system was carefully pulled out of the soil, washed, dried, weighed, and scanned with a flatbed scanner at a resolution of 800 dpi and both the above- and belowground biomasses were dried at 70°C for 48 h and weighed as the total biomass (g). Finally, RhizoVision Explorer was used to measure root length (cm) (Seethepalli et al., 2021), which was used to calculate the SRL (cm/g), expressed as root length divided by the root dry mass.

Finally, the soil samples were mixed together within each treatment and air-dried to measure the soil nutrient content SOC and STN, which reflect soil fertility, soil quality, and consequently plant growth (Crowther et al., 2016). SOC was determined using a modified Walkley and Black wet oxidation method (Nelson et al., 1996) and STN by a modified macro-Kjeldahl digestion method (Bremner and Mulvaney, 1983); both were expressed as g/kg soil.

### 2.3 Statistical analyses

Firstly, we calculated the PSF as an index biomass data as follows:


$$PSF=\;\frac{O-\;F_{average}}{F_{average}}$$

where *O* is the biomass produced by species in the soil-feedback phase when grown in soil conditioned by the same species and *F_average_* is the average biomass produced in the soil-feedback phase when species are grown in soil conditioned by other species. These calculations were used as recommended by previous studies (Pernilla Brinkman et al., 2010; Forero et al., 2021).

Secondly, we used two-way analysis of variance (ANOVA) to test the effect of soil conditioning by native, invasive, and mixed species and control and soil feedback by native and invasive species on 1) the soil nutrient availability (SOC and STN) and PSF index and 2) the performance and productivity of native and invasive species (plant height, SLA, N_mass_, C_mass_, SRL, and total biomass). In both models, the soil parameters of different pots and traits or productivity on the level of individuals were dependent variables and soil conditioning (control, native, invasive, and mixed), and soil feedback (native and invasive) were used as explanatory factors.

Finally, to support the interpretation of the data, we performed pairwise comparisons using Tukey’s *post-hoc* test to determine if there are differences between native and invasive species under the four different soil-conditioning treatments (control, native, invasive, and mixed) for all the measurements. All statistical analyses were performed using R, version 4.3.0 (R Development Core Team, 2023); package “*stats*” used for ANOVA, and package “*rstatix*” was used to perform Tukey’s pairwise comparison (Kassambara, 2023).

## 3 Results

Overall, the results of the current study showed significant effects of different soil-conditioning treatments (by native, invasive, and mixed species and control) and soil feedback by native and invasive species on SOC, STN, total biomass, plant height, SLA, N_mass_, C_mass_, and SRL (**Table 1**).

**Table 1:** Results of two-way ANOVAs testing the effects of soil conditioning by native only, invasive only, mixed “native and invasive” species and control “unconditioned soil” and feedback by native *P. cineraria* and invasive species *P. juliflora* on the soil parameters, SOC and STN, and on the production and performance of the native species *P. cineraria* and invasive species *P. juliflora*, total biomass, plant height, SLA, leaf nitrogen content (N_mass_), leaf carbon content (C_mass_), and SRL.

	Species origin in condition phase		Species origin in feedback phase	
	*F*	*P*	*F*	*P*
1. Soil parameters and PSF:				
SOC	29.24	<0.001	12.01	<0.001
STN	14.99	<0.001	4.71	0.03
PSF index	44.68	<0.001	5.49	0.02
2. Plant productivity and performance:				
Biomass	64.88	<0.001	4.7	0.004
Plant height	12.06	<0.001	6.15	0.013
SLA	27.02	<0.001	39.37	<0.001
N_mass_	14.778	<0.001	7.35	0.007
C_mass_	18.15	<0.001	13.16	<0.001
SRL	77.36	<0.001	21.58	<0.001

### 3.1 Effects of different soil-conditioning treatments and feedback on soil parameters and plant–soil feedback index

**Figure 2:**
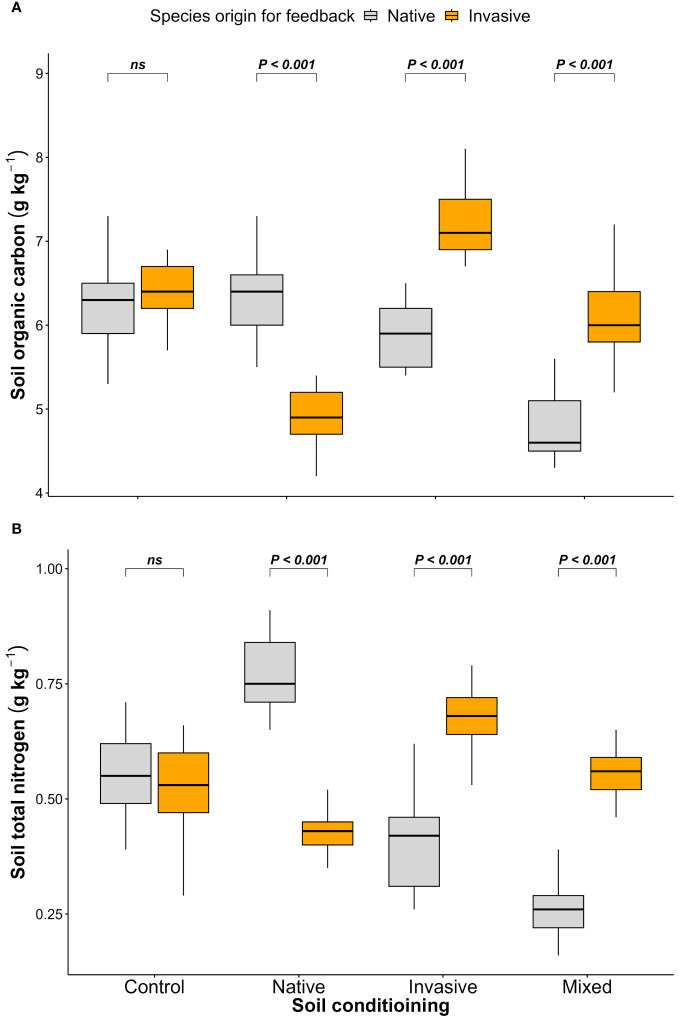
Effect of soil conditioning by control “unconditioned soil” and native, invasive, and mixed “native and invasive” species and feedback by native *P. cineraria* (orange) and invasive species *P. juliflora* (gray) on the soil parameters **(A)** SOC and **(B)** STN (*n* = 10 pots per treatment in the soil-conditioning phase and *n* = 5 pots per treatment per species in the feedback phase). *P*-values stand for statistically significant differences between native and invasive species based on pairwise comparisons using Tukey’s multiple comparison test (*ns*: non-significant differences).

The results of the feedback phase showed significant differences between the native (*P. cineraria*) and invasive species (*P. juliflora*) in terms of soil parameters (SOC and STN) under soil-conditioning treatments (native, invasive, and mixed). There were no significant differences between native and invasive species in terms of SOC and STN in the unconditioned soil (control) treatment (**Figures 2A, B**; **Table 1**). While the native species (*P. cineraria*) showed lower SOC and STN values in comparison to the invasive species (*P. juliflora*) by 23.4% and 65.6% in soil conditioned by invasive species and by 28% and 109% under soil conditioned by mixed species, it had higher values for both SOC and STN by 23.3% and 43.2% in soil conditioned by native species (**Figures 2A, B**; **Table 1**).

**Figure 3:**
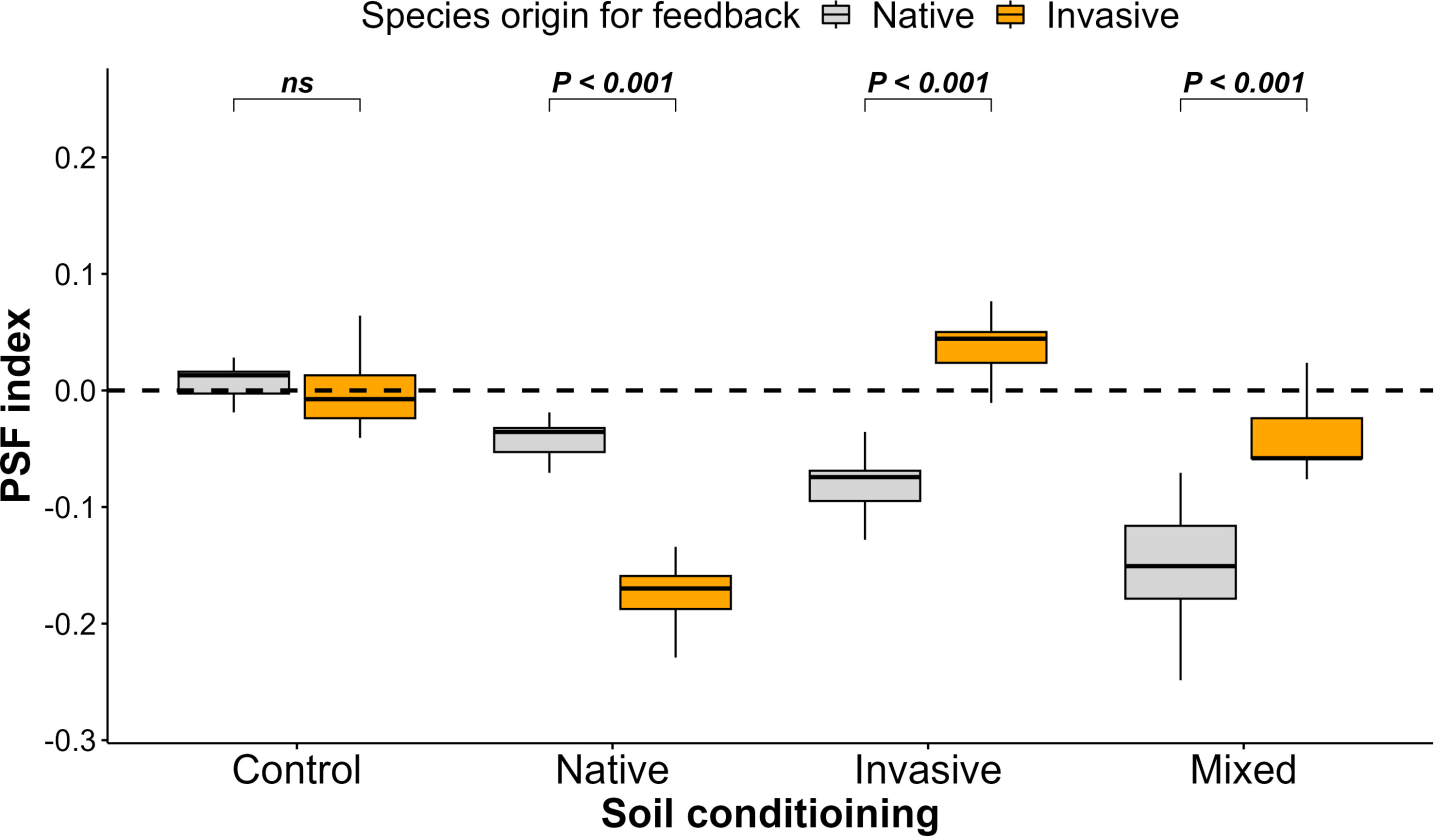
Effect of soil conditioning by control “unconditioned soil” and native, invasive, and mixed “native and invasive” species and feedback by native and invasive species on the plant–soil feedback index of the native species *P. cineraria* (orange) and invasive species *P. juliflora* (gray) (*n* = 10 pots per treatment in the soil-conditioning phase and *n* = 5 pots per treatment per species in the feedback phase). *P*-values stand for statistically significant differences between native and invasive species based on pairwise comparisons using Tukey’s multiple comparison test (*ns*: non-significant differences).

Concerning the PSF index, we noticed a negative PSF for native species grown in soil conditioned by invasive and mixed species, a positive PSF for invasive species grown in soil conditioned by invasive species, and a neutral PSF among native and invasive species in the control treatment (**Figure 3**).

### 3.2 Effects of different soil-conditioning treatments and feedback on the productivity and performance of invasive and native species

**Figure 4:**
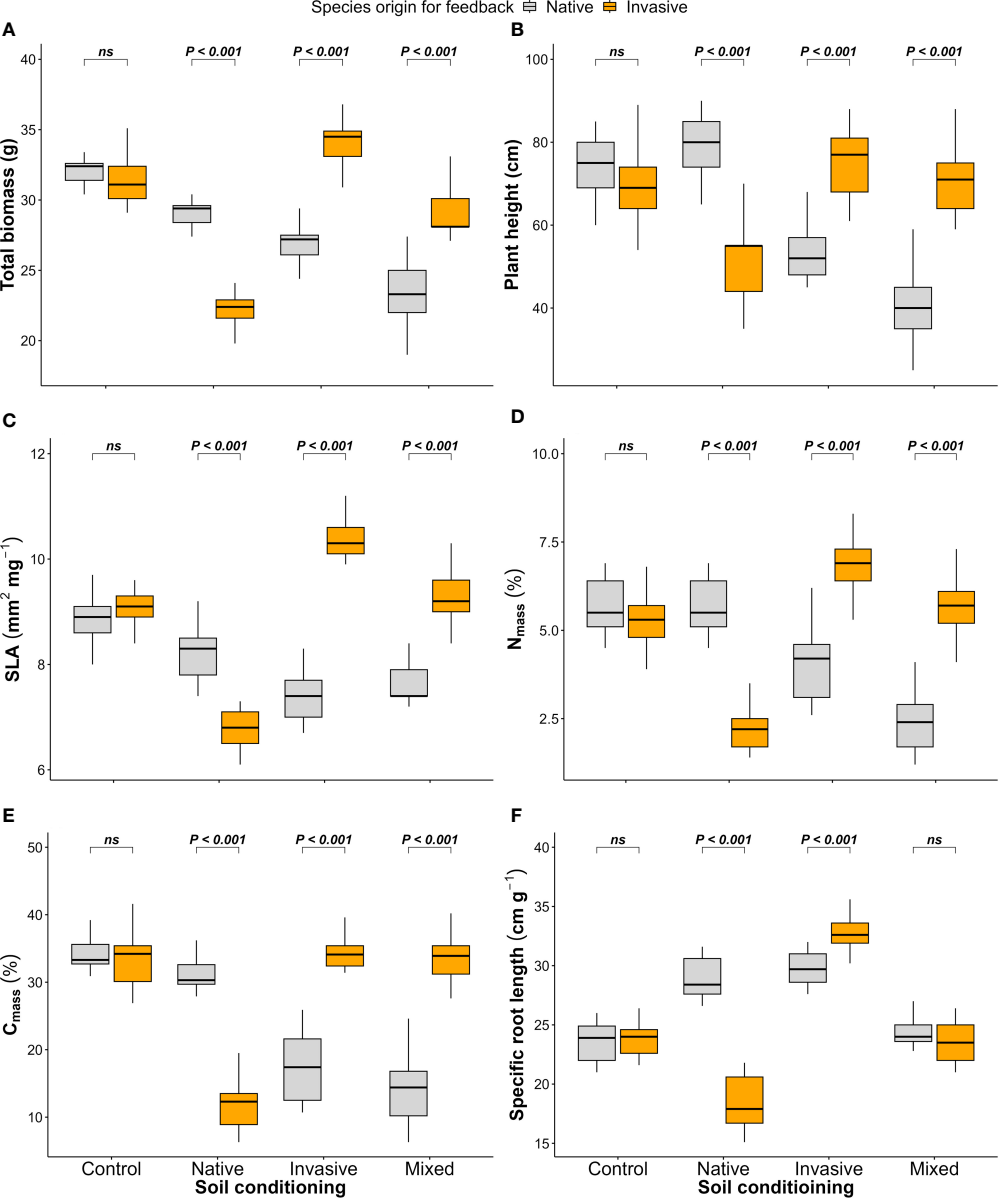
Effect of soil conditioning by control “unconditioned soil” and native, invasive, and mixed “native and invasive” species and feedback by native and invasive species on the production and performance of the native species *P. cineraria* (orange) and invasive species *P. juliflora* (gray): **(A)** total biomass, **(B)** plant height, **(C)** SLA, **(D)** leaf nitrogen content (N_mass_), **(E)** leaf carbon content (C_mass_), and **(F)** SRL (*n* = 10 pots per treatment in the soil-conditioning phase and *n* = 5 pots per treatment per species in the feedback phase). *P*-values stand for statistically significant differences between native and invasive species based on pairwise comparisons using Tukey’s multiple comparison test (*ns*: non-significant differences).

The native (*P. cineraria*) and invasive species (*P. juliflora*) showed significant differences in terms of total biomass under soil-conditioning treatments (native, invasive, and mixed) and no significant difference under unconditioned soil (control) treatment (**Figure 4A**; **Table 1**). We found that the native species (*P. cineraria*) produced less biomass when compared to the invasive species (*P. juliflora*) in soil conditioned by invasive and mixed species by 27.7% and 23.1%, respectively, and higher biomass by 24.2% in soil conditioned by native species (**Figure 4A**; **Table 1**).

Concerning species performance, the native species (*P. cineraria*) had significantly lower plant height, SLA, N_mass_, C_mass_, and SRL by 38.7%, 40.4%, 72.9%, 99.2%, and 9.5% in comparison to the invasive species (*P. juliflora*) when grown in soil conditioned by invasive species and significantly higher values for plant height, SLA, N_mass_, C_mass_, and SRL by 34.4%, 18.1%, 60.3%, 60.8%, and 35.9% when grown in soil conditioned by native species only (**Figures 4B–F**; **Table 1**). Additionally, the difference between the native (*P. cineraria*) and invasive species (*P. juliflora*) in soil conditioned by mixed treatment was significant for plant height, SLA, N_mass_, and C_mass_ as the native species (*P. cineraria*) showed significantly lower plant height, SLA, N_mass_, and C_mass_ by 80.1%, 21.3%, 138.2%, and 136.7%, and this difference was not significant in SRL (**Figures 4B–F**; **Table 1**). The soil conditioning and feedback phases affected species performance (plant height, SLA, N_mass_, C_mass_, and SRL) significantly as shown by ANOVA results (**Table 1**).

## 4 Discussion

The current study is one of the few studies investigating the factors that control the successful spread of the invasive plant species *P. juliflora* using the plant functional trait approach. We showed that that soil conditioned by invasive and mixed species improved the productivity and performance of the subsequent invasive species; in contrast, soil conditioned by native species enhanced the productivity and performance of the subsequent native species. There was no significant difference in plant productivity and performance between native and invasive species in the unconditioned soil “control.” These results suggest that when soil was conditioned by invasive species, this resulted in a negative PSF for the subsequent native species and a positive PSF for the subsequent invasive species. Moreover, when the soil was unconditioned, this resulted in a neutral PSF for the subsequent native and invasive species (Simberloff and Von Holle, 1999; Zhang et al., 2020; Chen and van Kleunen, 2022a). Negative PSF results from the ability of invasive species to change soil nutrients during the conditioning phase or the ability of invasive species to produce allelopathic chemicals that limit the growth of subsequent natives (De Long et al., 2023), positive PSF is due to the superior competition ability of the invasive species to utilize soil nutrients in comparison to the native species (Zhang et al., 2022), and neutral PSF suggests that both species have comparable soil nutrient requirements as they grow in soil that was not conditioned by any species (Cushman and Gaffney, 2010; Oschrin and Reynolds, 2020). Moreover, this confirms the “invasional meltdown” hypothesis, which suggests that the presence of invasive species can promote the establishment of the same or new invasive species (Simberloff and Von Holle, 1999; Zhang et al., 2020).

### 4.1 Effects of different soil-conditioning treatments and feedback on soil parameters

Both SOC and STN are considered key elements in assessing soil fertility and can be affected by several biotic and abiotic factors (Crowther et al., 2016; Gibbons et al., 2017); in turn, SOC and STN will affect several functional processes in soil related to water-holding capacity and soil aggregate stability (Murphy, 2015). In the current study, higher SOC and STN were found in the soil conditioned by native species and lower SOC and STN when the soil was conditioned by invasive and mixed species. These results go in line with previous research (Wang et al., 2018; Ali and Bucher, 2022; Zhang et al., 2023) confirming that invasive plants can also affect the N and C cycles in the soil by affecting soil microbial communities (Gibbons et al., 2017) and arbuscular mycorrhiza fungi (AMF) (Vogelsang and Bever, 2009; de Souza et al., 2018) due to the allelopathic substances released by invasive species (Torres et al., 2021). These results suggest that alteration in the soil nutrient content by invasive species is vital to facilitate invasion success (Blackburn et al., 2011) in a way to compete with native species (Čuda et al., 2015).

### 4.2 Effects of different soil-conditioning treatments and feedback on the productivity and performance of invasive and native species

In the current study, as the native species performed better than the invasive species in terms of plant functional traits when the soil was conditioned by native species and the contrary was found under soil conditioning by invasive or mixed species, this suggests that plant functional traits can explain the changes in PSF due to the presence of native and invasive species (De Long et al., 2023).

The higher biomass of *P. cineraria* compared with that of *P. juliflora* when the soil was conditioned by native species and the lower biomass when the soil was conditioned by invasive and mixed species are in agreement with previous studies (Zhang et al., 2020; Chen and van Kleunen, 2022b), confirming the ability of the invasive species to change the soil microorganisms and consequently the PSF (Zhang et al., 2020). This effect was not obvious when the soil was conditioned by native species due to the lack of invasives, which is in line with other studies (Hohenadler et al., 2018; Shen et al., 2023) that show the ability of invasive species to promote the growth of subsequent invasive species.

*P. cineraria* grew larger than *P. juliflora* in soil conditioned by native species, suggesting that the native plant competed more strongly for resources when grown in soil conditioned by a native species (Closset-Kopp et al., 2011) and consequently improved nutrient acquisition (Moles et al., 2009). On the other hand, it grew smaller than the invasive species in soil conditioned by invasive and mixed species, which suggests that the invasive species got benefits of the altered PSF due to the presence of previous invasive species (De Long et al., 2023). The same trend was found for SLA, suggesting that species with a high SLA reduce the performance of other species due to the altered PSF (Fitzpatrick et al., 2017). Moreover, that the native species got a higher SLA in the soil conditioned by native species and a lower SLA in the soil conditioned by invasive and mixed species can be explained as being due to the fact that SLA reflects the plant growth rate and nutrient acquisition strategy (Wright et al., 2004), which confirms the superiority of invasive species in nutrient acquisition when conditioning soil in comparison to the native one (Gommers et al., 2013; Ali et al., 2023).

It was reported that N_mass_ and C_mass_ in leaves are correlated with the availability of STN and SOC and the abilities of plants to make use of them (Elser et al., 2007), which was reflected in our results as *P. cineraria* had higher N_mass_ and C_mass_ than the invasive *P. juliflora* when the soil was conditioned by native species and lower N_mass_ and C_mass_ when the soil was conditioned by invasive and mixed species. These results indicate that native species can effectively utilize the available STN and SOC in the soil when no competition found from previous invasive species, but when the invasive species present in the soil prior to the native species, it gives the superiority to the invasive species P. juliflora to utilize these resources more efficiently (Ehrenfeld, 2003; Hu et al., 2019). Interestingly, *P*. *cineraria* showed lower values for total biomass, plant height, N_mass_, C_mass_, SRL, SOC, STN, and PST index when grown in soil conditioned by mixed species than when grown in soil conditioned by invasive species. These results suggest that the invasive *P. juliflora* is a successful invader not only due to the changes it creates in its soil environment but also as a result of its competitive ability (Perkins and Nowak, 2012).

## 5 Conclusion and recommendations

The current study revealed a positive PSF for the invasive species *P. juliflora* and a negative PSF for the native species *P. cineraria*, which can be an alarming sign for conservation and restoration of *P. cineraria* as an important native plant in Oman. These results suggest that the presence of *P. juliflora* will create a barrier to the reintroduction and establishment of native species like *P. cineraria*. In addition to these findings, it is important to emphasize the pursuance of further research into the underlying mechanisms of *P*. *juliflora*’s successful invasion. Specially, exploring the role of AMF in its interactions with soil microorganisms and delving into the allelopathic activities of *P*. *juliflora* can provide a more comprehensive understanding of its impact on the local ecosystem. Furthermore, understanding the complex relationships between *P*. *juliflora* and these soil components is applicable not only for managing its aggressive spread but also for formulating more effective tactics for preservation and restoration of native species like *P*. *cineraria* in Oman. Such insights can inform targeted interventions to mitigate the adverse effects of invasive species and facilitate the resurgence of vital native plants, ultimately contributing to the preservation of Oman’s unique ecological balance.

## Data availability statement

The original contributions presented in the study are included in the article/**Supplementary Material**. Further inquiries can be directed to the corresponding author.

## Author contributions

HA: Conceptualization, Data curation, Formal analysis, Funding acquisition, Investigation, Methodology, Project administration, Resources, Supervision, Validation, Visualization, Writing – original draft, Writing – review & editing. AA-W: Methodology, Project administration, Writing – review & editing. MS: Methodology, Writing – review & editing.
