# Supplementary material for: Plant–soil feedback and plant invasion: effect of soil conditioning on native and invasive Prosopis species using the plant functional trait approach
Source: Front Plant Sci. 2024 Jan 16;15:1321950. doi: 10.3389/fpls.2024.1321950 (PMC10824832; doi:10.3389/fpls.2024.1321950)
Supplement: Supplementary file 1 [file DataSheet_1.docx]

Supplementary Material

## Supplementary Figures


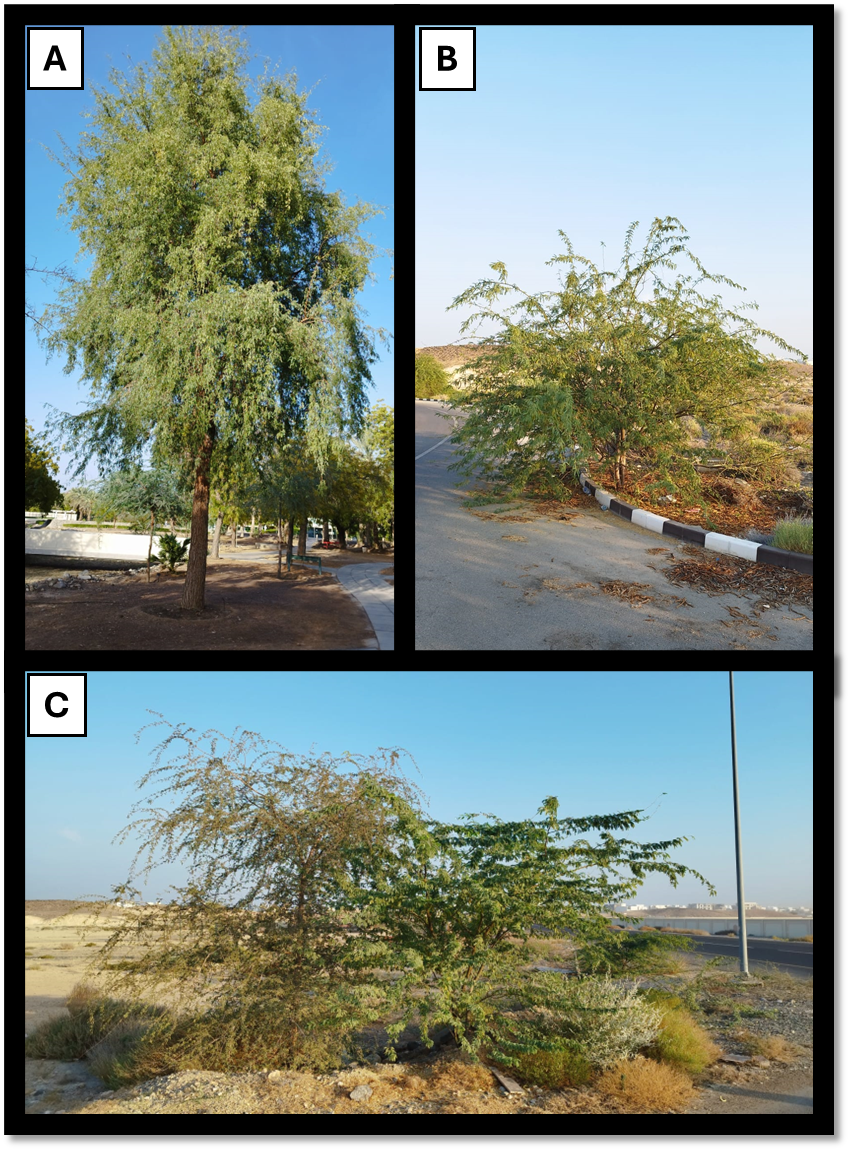


**Supplementary Figure 1.** Studied species in their natural habitat, (A) *Prosopis cineraria*, (B) *Prosopis juliflora,* and both species growing together left is *Prosopis cineraria* and right is *Prosopis juliflora*.
